# Handheld Thermal Devices Can Facilitate Population Monitoring of the Critically Endangered Delacour's Langur *Trachypithecus delacouri* in Difficult Terrains

**DOI:** 10.1002/ece3.73057

**Published:** 2026-02-04

**Authors:** Anh Tuan Nguyen, Linh Nguyen, Hoang Trinh‐Dinh, Phong Nguyen, Thanh Nguyen, Minh Le

**Affiliations:** ^1^ Faculty of Environmental Sciences University of Science, Vietnam National University Hanoi Vietnam; ^2^ Southern Institute of Ecology Ho Chi Minh City Vietnam; ^3^ Conservation Ecology Program, School of Bioresources and Technology King Mongkut's University of Technology Thonburi Bangkok Thailand; ^4^ Save Vietnam's Wildlife Cuc Phuong Vietnam; ^5^ Faculty of Biology University of Science, Vietnam National University Hanoi Vietnam; ^6^ Central Institute for Natural Resources and Environmental Studies Vietnam National University Hanoi Vietnam

**Keywords:** Kim Bang, population survey, thermal device, *Trachypithecus delacouri*, Vietnam

## Abstract

The Delacour's langur ( *Trachypithecus delacouri* ) is a Critically Endangered primate, restricted to a small region in northern Vietnam. In view of its very small population and existing threats, frequent population monitoring programs are urgently needed for this species. In this study, we evaluated the utility of handheld thermal imaging devices as a complementary tool to conventional ground‐based visual surveys for primate population monitoring efforts. Based on results of past studies, we conducted field surveys in Kim Bang Protection Forest, Ninh Binh Province, Vietnam, where the second most important population of the Delacour's langur inhabits. While we followed protocols from previous ground‐based visual surveys, we also used thermal monoculars to facilitate langur detections. By integrating thermal handheld devices, we documented at least 18 langur groups with around 116 individuals, an increase of about 11.5% in total population size compared to the most recent extensive study at the same site. When comparing to the 2022 drone survey in Kim Bang, our results also showed that while the drone platform demonstrates superior performance, the integration of thermal imaging devices substantially reduces survey effort relative to conventional ground‐based visual techniques. Given the recent regulations on flying drones in remote areas in Vietnam, our findings suggest that thermal imaging devices offer a viable option to improve the efficacy of ground‐based primate population monitoring surveys. Furthermore, when properly deployed, handheld thermal devices may provide key advantages for certain primate research topics.

## Graphical abstract

This study demonstrates that handheld thermal devices can effectively complement traditional ground‐based surveys for monitoring the critically endangered Delacour's langur in northern Vietnam. Fieldworks also revealed a population increase and reduced survey effort, highlighting thermal handhelds as a practical alternative amid drone flight restrictions.

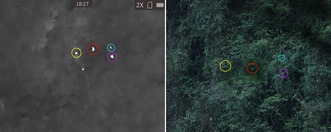

## 1 Introduction

The Delacour's langur ( *Trachypithecus delacouri* ) is a Critically Endangered primate currently found only in a restricted rugged limestone landscape of northern Vietnam (Nadler et al. 2020). Since the late 1980s, the langur populations have plummeted because of illegal hunting and habitat loss. Consequently, from the early 2000s through the mid‐2010s, the Delacour's langur was consistently listed among the 25 most endangered primates in the world (Schwitzer et al. 2015). Recent studies estimated that fewer than 550 individuals of the Delacour's langur remain in the wild, and a majority of them occupy just three locations: Van Long, Kim Bang, and Yen Mo, all situated in Ninh Binh Province (Linh et al. 2019; Nadler et al. 2020; Trinh‐Dinh, Giang, et al. 2024; Trinh‐Dinh, Wearn, et al. 2024; Trinh‐Dinh, unpublished data, 2025). Among those, Kim Bang Protection Forest supports the second largest extant population of the species with about 104 animals (Trinh‐Dinh, Wearn, et al. 2024).

For a severely threatened species such as the Delacour's langur, frequent population monitoring is crucial to inform and guide effective conservation activities. However, the implementation of such surveys has been constrained by a number of reasons, such as limited financial resources for extensive field surveys and scarce access to its habitat. One particular challenge for the Delacour's langur monitoring efforts is that their forested limestone habitats, which are characterized by steep, heavily eroded, and difficult‐to‐access terrains, pose substantial obstacles for ground‐based surveys (Nguyen et al. 2022; Trinh‐Dinh, Wearn, et al. 2024). Recently, different approaches have been explored to partly address such issues, including drone‐based surveys (Gazagne et al. 2025, 2023; Karp 2020; Wearn, Trinh‐Dinh, Le, and Nguyen 2024). Although highly effective, the drone‐based primate surveys in Vietnam have several drawbacks, such as short flight time, high initial acquisition cost, and regulatory changes (Trinh‐Dinh, Wearn, et al. 2024). For instance, according to a regulation that went into effect in 2025, all non‐recreational drone operations are required to submit detailed flight plans and other supporting documents for prior approval by relevant authorities (The National Assembly of Vietnam 2024). Also, extensive restricted‐fly zones, where acquiring permissions might be considerably protracted, have been designated across the country. Those zones encompass about one‐third of Kim Bang and half of Yen Mo Protection forests (The National Assembly of Vietnam 2024), both of which are critical habitats for the Delacour's langur populations.

Other approaches that offer improved performance over conventional ground‐based visual surveys for monitoring endangered primate populations, yet do not face similar logistic and legal constraints of drone surveys, are therefore urgently needed. An essential advantage that has been emphasized in recent primate surveys is the integration of thermal imaging devices with other platforms (Trinh‐Dinh, Wearn, et al. 2024; Wearn, Trinh‐Dinh, Le, and Nguyen 2024). Standalone thermal imaging devices have been widely used to detect and record mammals, as individual animals can be highlighted against the surrounding backgrounds, and hence it gives crucial clues to researchers for finding and estimating populations in the field (Cilulko et al. 2013). The benefits from the use of handheld thermal devices in assisting ground‐based surveys for arboreal mammals, especially for primate taxa, have also been confirmed in previous studies (Jumail et al. 2021; Pocknee et al. 2021; Vinson et al. 2020). Handheld thermal devices are also more affordable than drones and do not require specialized training. As such, they represent a viable option for Vietnam's small‐ to medium‐sized non‐governmental organizations and local authorities, who often have to operate under constrained resources and technical capacities (Appleton et al. 2022; Nguyen et al. 2025; Pocknee et al. 2021).

In addition, given the recent regulatory developments in Vietnam, handheld thermal imaging devices may offer a practical and cost‐effective alternative that can significantly improve primate ground‐based surveys. To evaluate the efficacy of such devices in complementing conventional surveys for the Delacour's langur population monitoring, we conducted field surveys in Kim Bang Protection Forest, Ninh Binh Province, in April and July 2025 using both visual observations and thermal devices. We then compared our results with previous conventional ground‐based and drone surveys of the Delacour's langur at the same site.

## 2 Materials and Methods

### 2.1 Study Area

Kim Bang Protection Forest (KBPF) is located in Ninh Binh Province, Vietnam, and managed by local authorities. It is a watershed protection forest and not listed in the national protected area network. As KBPF only has limited legal protection, it has experienced extensive exploitation of natural resources, especially logging and limestone mining, over several decades (Nguyen et al. 2025, 2022; Trinh‐Dinh, Wearn, et al. 2024). Despite severe habitat degradation, KBPF is home to the second largest population of the Delacour's langur and has been recommended to be elevated to the protected area status since 2019 (Nguyen et al. 2022; Thanh 2020). The proposed nature reserve covers an area of around 3100 ha. The core zone of approximately 1800 ha, where almost all langur groups inhabit, is characterized by highly dissected limestone karst formations and narrow valleys. This area is covered with thick, dense limestone vegetation composed of shrubs, small trees, and lianas. Active limestone quarrying operations, which demolish the critical habitat of the Delacour's langur, take place along the northern, eastern, and southern sides of KBPF (Nguyen et al. 2022; Trinh‐Dinh, Wearn, et al. 2024).

KBPF is located in a humid subtropical climate zone, and the local climate shows significant variations around the year, especially in temperature, sunlight hours, and precipitation (Trinh‐Dinh, Wearn, et al. 2024). During our April survey, on average, the lowest and highest daily temperatures were recorded at 22°C and 29°C, respectively, with the mean daily temperature of about 24°C. Sunrise and sunset were at about 05:40 h and 18:10 h, respectively. In contrast, during our July survey, on average, the lowest and highest daily temperatures were recorded at 27°C and 36°C, respectively, with the mean daily temperature of about 31°C. The sunrise and sunset were at about 05:30 h and 18:40 h, respectively.

### 2.2 Field Surveys

#### 2.2.1 Survey Optimizations

Before conducting the field surveys, we reviewed relevant publications on Kim Bang's langur population, especially from the 2018, 2020, and 2022 surveys, to identify potential survey areas and to select suitable locations for observation points (Nguyen et al. 2022; Thanh 2020; Trinh‐Dinh, Wearn, et al. 2024). We also interviewed five local people knowledgeable about KBPF and Delacour's langurs to validate our selections.

As the use of thermal devices to survey primates has been shown to be highly influenced by ambient temperature, we conducted two field surveys with identical protocol in two different seasons to better assess the performance variability of handheld thermal devices in different conditions. The first survey was conducted from late April to early May 2025, when the weather was still influenced by the late spring season, with relatively cool temperatures and shorter sunlight hours. The second survey was carried out from late July to early August, during the summer season, with relatively high temperatures and longer sunlight hours. Each survey lasted 10 days. Based on previous studies (Gazagne et al. 2024; Wearn, Trinh‐Dinh, Ma, et al. 2024), we assumed that the environmental conditions during the April survey would be more conducive to effective thermal detection of langurs. Accordingly, our population analysis focused on data obtained during the April fieldwork. In contrast, the July survey was conducted primarily to evaluate the operational performance of thermal imaging devices under elevated temperature conditions. As such, data from the July survey were excluded from population estimates and were referenced solely in the context of device effectiveness.

#### 2.2.2 Data Collection

**FIGURE 1:**
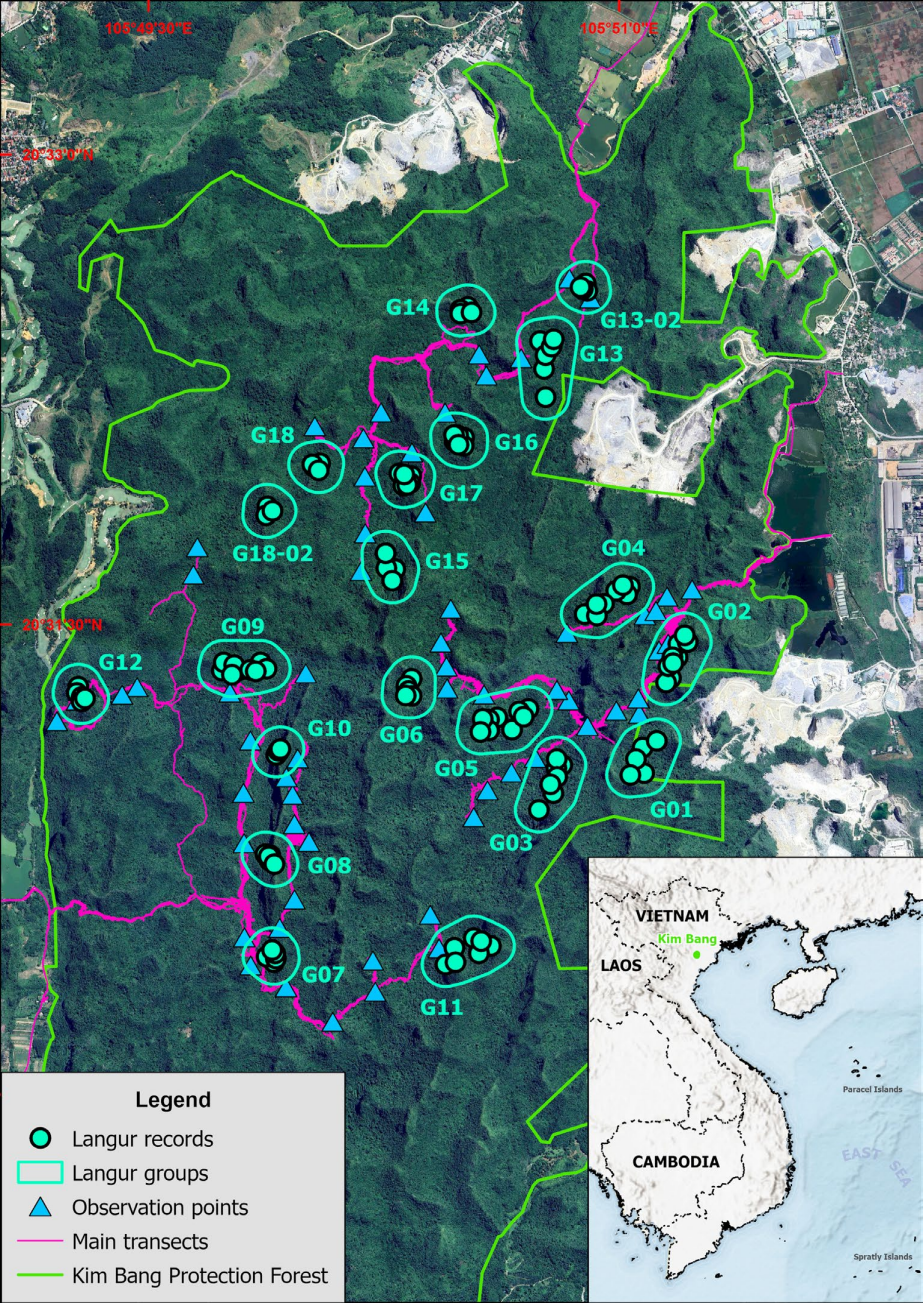
Survey locations and observations of Delacour's langur in our April survey in Kim Bang Protection Forest.

For field surveys, we used the general ground‐based approach that was successfully employed in the 2018 study (Nguyen et al. 2022), with several modifications to accommodate the complementary use of handheld thermal devices. In particular, in each survey, we deployed two field teams, and each field team included one researcher and one local assistant. To maximize detection probability and spatial coverage, each day, each team was responsible for independent observation points. As the langurs often have sentries that warn the groups of potential threats, and they have tendencies to retreat upon detecting approaching humans, we primarily selected observation points at elevated locations overlooking langurs' potential sleeping and feeding sites during their active hours (05:00–09:00 h and 16:00–20:00 h) to record the Delacour's langur. Additionally, we walked along existing transects to look for signs of possible langur groups and supplementary observation sites (Figure 1).

Each field team was equipped with an optical monocular (Endurance 10 × 40—Hawke Sport Optics, United States) and a handheld thermal monocular (TM19‐384—AGM Global Vision, United States) to facilitate langur detection. The AGM TM19‐384 was selected as it was a commercially available and affordable product. It featured a moderate 384 × 288 resolution, 12 μm vanadium oxide uncooled detector, and could digitally zoom in up to eight times. As advertised by manufacturers, both optical and thermal monoculars can provide visibility up to about 900 m under optimal conditions, suggesting comparable performance in terms of detection range.

At each observation point, a researcher conducted continuous thermal and visual scans to look for possible signs of the Delacour's langur, employing both thermal and optical monoculars to enhance the detection probability. Upon initial langur sightings, the researcher recorded time and date, coordinates of the observation point, directional bearing and estimated distance to the langurs, group size and structure, their behaviors and activities, and the specific device that facilitated the initial detection. The age–sex identification between individuals (adult male, adult female, juvenile, infant), whenever possible, followed recommendations from Nadler et al. (2003) and Workman (2010). To help assess the effectiveness of thermal imaging devices, during the observation, researchers documented the number of langurs detected and counted exclusively via optical monocular, as well as those identified through the combined use of both optical and thermal devices. Any discrepancies in detection capacities between the two types of monoculars were noted. Other important information, such as general weather conditions, ambient temperature, and habitat characteristics, was also recorded.

To minimize the risk of double counting langur groups that traveled extensively during the day, at the end of each day, the teams compared the time and location of langur encounters, as well as group size and group composition, to determine distinct groups. Any groups that could not be clearly distinguished were treated as a single group. When there were different counts for the same group, we kept both values. To compare the detection improvements between the visual‐only and thermal device‐assisted observations in our April survey, we used the Wilcoxon signed‐rank test for statistical significance, as the collected population data was not normally distributed (Jumail et al. 2021; Pöysä et al. 2018).

## 3 Results

### 3.1 Delacour's Langur Population Status in Kim Bang

Our April survey recorded at least 18 langur groups with a minimum of 116 individuals in KBPF (Figure 1, Table 1). We also recorded two male‐only groups with a total of four animals. Including male‐only groups, the total population of the Delacour's langur in KBPF would be around 120–121 individuals. Excluding male‐only groups, the mean group size was 6.44, with a standard deviation of 2.01. The maximum and minimum group sizes were 11 and four individuals, respectively, well within the known population metrics range of the species (Nguyen et al. 2022; Trinh‐Dinh, Wearn, et al. 2024). In total, 11 young individuals, including nine infants and two juveniles, were observed, accounting for around 9.5% of the total population. The handheld thermal devices, in complement with visual observations, showed significant improvement for langur individual identification, with approximately 56.7% more langur individuals on average than those documented using optical monoculars alone (*r* = 0.88, *p* < 0.01 for Wilcoxon signed‐rank test). Furthermore, at least six langur groups would have gone unrecorded entirely without the heat signatures provided by thermal scanning (Table 1). In comparison, our July survey documented seven langur groups, and thermal devices helped detect only two groups.

**TABLE 1:** Details of the Delacour's langur groups documented in our April survey in Kim Bang Protection Forest.

Group	Group size	Juveniles and infants	Detection note	Other note
G01	8	01 juvenile and 01 infant	Visual only: 6 Thermal + Visual: 8	Shared sleeping cave with G02.
G02	8–9	01 infant	Visual only: 8–9 Thermal + Visual: 8–9	Shared sleeping cave with G01.
G03	5	0	Visual only: 3 Thermal + Visual: 5	
G04	5	0	Visual only: 4 Thermal + Visual: 5	
G05	7	01 juvenile and 01 infant	Visual only: 5 Thermal + Visual: 7	
G06	6	0	First signs from thermal monocular scan only. Visual only: 2 Thermal + Visual: 6	
G07	6	01 infant	Visual only: 5 Thermal + Visual: 6	Shared sleeping cave with G08.
G08	7	0	Visual only: 5 Thermal + Visual: 7	Shared sleeping cave with G07.
G09	4	0	Visual only: 1 Thermal + Visual: 4	
G10	8	0	First signs from thermal monocular scan only. Visual only: 1 Thermal + Visual: 8	
G11	5	01 infant	First signs from thermal monocular scan only. Visual only: 3 Thermal + Visual: 5	
G12	4	0	Visual only: 3 Thermal + Visual: 4	
G13	7	0	First signs from thermal monocular scan only. Visual only: 4 Thermal + Visual: 7	
G13‐02	1	0	Visual only: 1 Thermal + Visual: 1	One male‐only group. Local assistants reported that it used to be a member of G13.
G14	4	0	Visual only: 4 Thermal + Visual: 4	
G15	9	01 infant	Visual only: 7 Thermal + Visual: 9	
G16	8	01 infant	First signs from thermal monocular scan only. Visual only: 1 Thermal + Visual: 8	
G17	4	0	Visual only: 4 Thermal + Visual: 4	
G18	11	0	First signs from thermal monocular scan only. Visual only: 8 Thermal + Visual: 11	
G18‐02	3	0	First signs from thermal monocular scan only. Visual only: 0 Thermal + Visual: 3	Male‐only group. Local assistants reported that at least one member of this group used to be a member of G18.
Total	116–117	02 juveniles and 09 infants	Visual only: 74–75 Thermal + Visual: 116–117	Exclude male‐only groups.
	120–121		Visual only: 75–76 Thermal + Visual: 120–121	Include male‐only groups.

### 3.2 Survey Method Comparison

To evaluate the relative efficiency of different survey approaches, we compared several key metrics of our results from the April survey with the 2022 and 2018 studies (Table 2). The 2022 survey was conducted using a specialized drone platform (Mavic 2 Enterprise Advanced) outfitted with dual optical and thermal imaging systems (Trinh‐Dinh, Wearn, et al. 2024), whereas the 2018 survey relied exclusively on conventional ground‐based visual observations without the aid of thermal imaging devices (Nguyen et al. 2022). In general, the drone‐assisted method demonstrated superior performance, as it offered the most extensive spatial coverage with the least time investment, and it also required minimal effort per langur group/individual detected. When comparing the thermal device‐assisted ground‐based approach utilized in the present study with the conventional ground‐based method of the 2018 survey, the effort required to achieve comparable area coverage was relatively similar. However, the thermal‐assisted approach required only 38%–44% of the survey hours needed by the conventional approach to detect a langur group/individual (Table 2). We acknowledge that the reduced effort needed by thermal‐assisted ground‐based surveys might be influenced by the informed site selection process based on previous studies prior to our field surveys. Together with more accurate population estimates, the findings highlight the significant improvement provided by thermal imaging devices in complementing conventional ground‐based surveys, particularly in challenging terrains.

**TABLE 2:** Comparisons between thermal device‐assisted ground‐based survey, 2022 drone survey, and 2018 conventional ground‐based survey approaches for the Delacour's langurs in Kim Bang Protection Forest.

No	Criteria	This study	2022 survey	2018 survey
1	No. of field teams	02	01	02
2	No. of field days	10	48	38
3	No. of survey hours	280	83	456
4	No. of langur groups recorded	18	16	13
5	No. of langur individuals recorded	116	104	73
6	% study site covered	59%	100%	72%
7	Survey hours needed/ha	0.210	0.037	0.280
8	Survey hours needed/langur group	15.56	5.19	35.08
9	Survey hours needed/langur individual	2.41	0.80	6.25

## 4 Discussion

**FIGURE 2:**
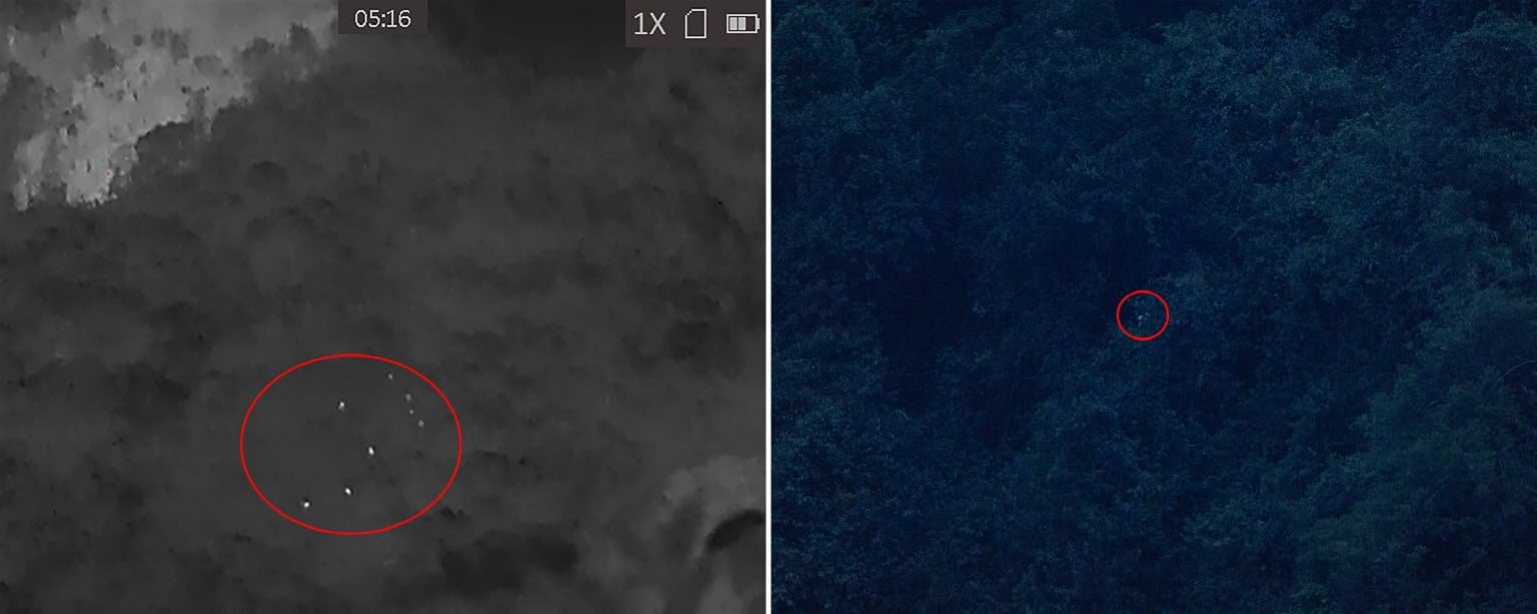
A Delacour's langur group's photos, taken by the thermal monocular (left, with eight animals indicated) and a normal digital camera (right, with only one animal barely visible) in very early morning time. This group would have been missed entirely had the researcher not scanned the area with a thermal device.

In this survey, while we use both optical and thermal monoculars to facilitate the detection of the Delacour's langur, the handheld thermal device is demonstrated to be considerably more effective in a number of circumstances, especially during the April survey. The first advantage of the thermal monocular is its capacity to extend observational windows when ambient light was limited, such as pre‐dawn (i.e., before around 05:45 h) and post‐sunset (i.e., after around 18:00 h) periods, when optical devices proved inadequate due to low visibility. Using thermal devices, we successfully identified and monitored langur group activities as early as 04:30 h and as late as 20:00 h. Those temporal extensions are particularly valuable, as Delacour's langurs exhibited heightened movements and social interactions during crepuscular periods, thereby increasing their detectability. Consequently, the integration of the thermal imaging device enables census efforts to be conducted well beyond the limits of ground‐based visual surveys (Figure 2).

**FIGURE 3:**
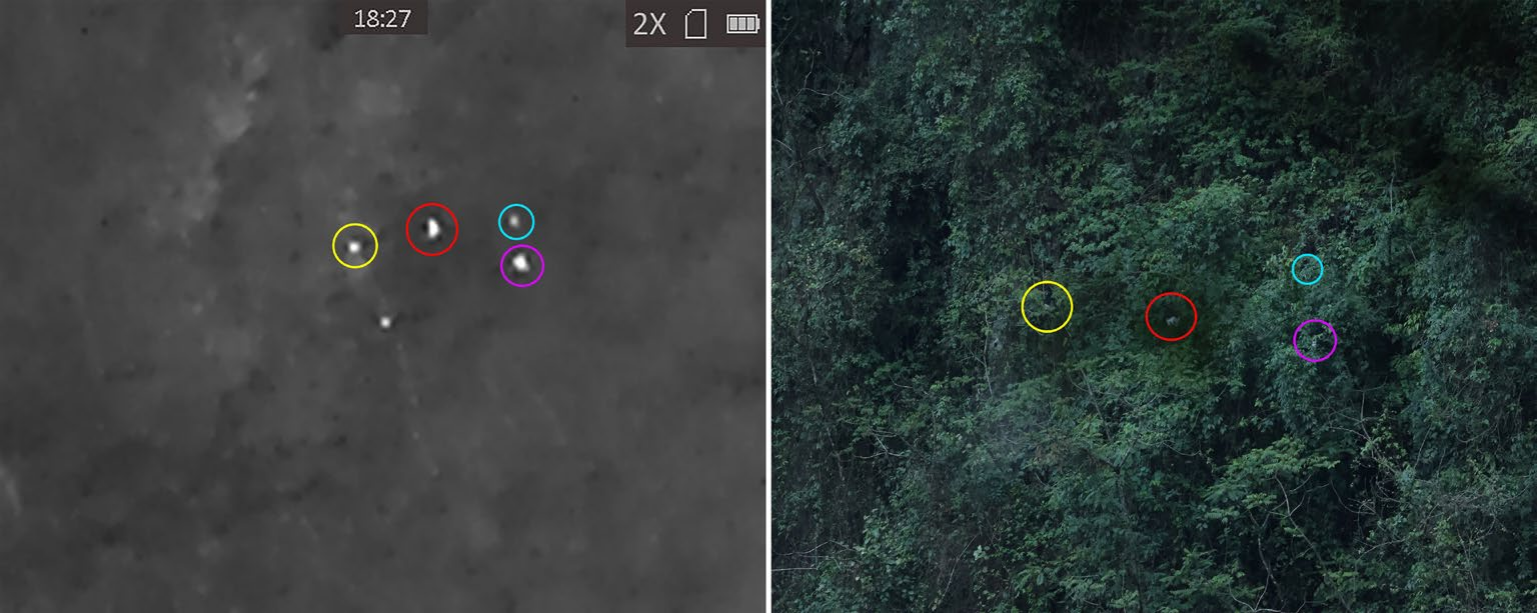
A langur group with only one member (red) fully visible in the right panel, and other animals were partly covered by canopy in proximity. The thermal monocular helped identify their positions for visual verifications.

The second advantage of the thermal devices is that, even in ample sunlight conditions, they may still offer better accuracy for detecting langur groups and counting langur individuals. During the period of feeding or resting, while a few animals may position themselves on exposed upper branches, many others remain concealed within lower strata or dense canopy cover, making visual detection difficult. However, thermal monoculars allow the detection of those hidden langur individuals by revealing their heat signatures against the cooler background, thereby facilitating subsequent visual confirmations. As a result, the combination of optical and thermal monoculars substantially augmented researchers' ability to locate and accurately count langur groups/individuals. As detailed in the result section, during the April survey, thermal devices provided critical cues that led to the identification of a significantly higher number of langur individuals (Figure 3). Those findings emphasize the value of integrating thermal imaging devices into ground‐based primate monitoring surveys, particularly in habitats characterized by dense vegetation structures and cover.

The third advantage of handheld thermal devices, particularly when compared to drone‐based surveys, is that as they are rechargeable through power banks, they allow for extended observation periods in each langur encounter. Prolonged observations can be highly beneficial for studying primate activities and behaviors, as they facilitate the documentation of infrequent or atypical behaviors. For example, in this study, extended observations from thermal devices recorded several instances in which distinct langur groups, arriving from different directions, converged upon and shared the same cave system for sleeping during the night (Table 1). This behavior is considered uncommon by previous studies of the Delacour's langur, and most previous research suggests that for members of the *T. francoisi* species complex, while sharing sleeping sites may occasionally take place, such events are typically temporally segregated (Hendershott 2017; Huang et al. 2003; Workman 2010). Our results highlight the potential of thermal imaging devices in uncovering novel aspects of the Delacour's langur behavioral ecology and their utility in future studies of social engagements and spatial cooperations within this species.

On the other hand, we also recognized several disadvantages of thermal devices when compared to conventional visual observation surveys. Firstly, consistent with findings from previous research (Trinh‐Dinh, Wearn, et al. 2024), the thermal monocular exhibited limited capacity to separate between age and sex classes within the Delacour's langur. Moreover, due to the physical proximity of infants to their mothers, thermal monoculars regularly rendered those individuals as a single undifferentiated heat unit, thereby reducing the accuracy of langur population assessments. Hence, visual confirmation remained essential for group composition recognition and population estimates.

The second disadvantage is the diminished performance of thermal monoculars under elevated ambient temperatures (Gazagne et al. 2023; Rahman et al. 2025). During the July survey, when we repeated the study under summer conditions, the same devices and protocol yielded markedly reduced detection capabilities. In total, only seven langur groups were recorded, with the majority identified through visual cues; thermal monoculars contributed to the detection of only two groups. We suggest it was likely attributable to sustained high temperatures throughout the day (daily mean = 31°C, daily high = 36°C), compounded by the thermal properties of the limestone karsts, which absorb sunlight during the day and retain heat well into the evening. Consequently, the optimal window for thermal‐assisted detections was confined to a brief period in the early morning. In contrast, during the April survey, thermal monoculars remained effective up to an ambient temperature of about 26°C. The use of thermal devices during cooler months, ideally when highest daily temperatures do not exceed 25°C, and the adoption of more advanced thermal technologies with higher sensitivity may mitigate such limitations.

Overall, the use of handheld thermal devices can provide a number of practical advantages over drone‐based surveys. As handheld thermal devices, similar to those presented in this study, are often compact, lightweight, and affordable, their use is more logistically feasible in remote regions. We also note that due to drones' battery and weather requirements, in many cases they could only fly for a few hours a day, which in turn might significantly increase the total survey days (Table 2). More importantly, unlike drones, handheld thermal devices are not subject to regulatory constraints such as pilot certification or pre‐approved flight plans, allowing further flexibility across regions. According to the new drone regulation (The National Assembly of Vietnam 2024), we estimated the total area of restricted fly zones in Vietnam to be more than 65,000 km^2^, encompassing part of at least 40 protected areas, approximately 22% of Vietnam's total protected areas. Many important sites for remaining endangered primate populations, such as Trung Khanh, Pu Mat, Vu Quang, Song Thanh, and Chu Mom Ray (Covert et al. 2017; Wearn et al. 2022, 2023), are either entirely or partially situated within such zones. Therefore, while handheld thermal devices may not match the capabilities and performances of drones in surveying primates, they represent a viable and cost‐effective alternative tool for primate population monitoring efforts at the sites.

In this study, despite a substantially shortened duration of fieldwork and a more limited spatial coverage compared to those in the 2018 survey, we were able to record more langur groups and individuals (Table 1). While the advantages provided by thermal monoculars are instrumental in this study, the improved results could also reflect benefits from survey optimization. Specifically, we used results from previous studies to concentrate field activities in the northern and central sectors of KBPF, known to harbor langur subpopulations. The handheld thermal devices may not provide the same effectiveness for field surveys that are more ‘exploratory’, that is, detecting unknown populations in unknown areas. Nevertheless, for primate populations that inhabit relatively isolated and fragmented sites and have been reasonably well‐surveyed (Linh et al. 2019; Tran et al. 2017; Ulibarri 2013; Wearn, Trinh‐Dinh, Ma, et al. 2024), handheld thermal devices offer significant improvements compared to the traditional methods.

The population size of the Delacour's langur in KBPF from this survey slightly increases compared to that estimated by the 2022 survey, which represented the most recent comprehensive monitoring effort in KBPF. While the 2022 survey only directly documented 16 groups and 104 langur individuals, the population estimation model based on the field study results extrapolated 25 groups and 175 animals for the entire KBPF (Trinh‐Dinh, Wearn, et al. 2024), higher than our individual counts. It is plausible that we were able to spot a few langur groups and individuals that the 2022 survey might have missed. However, it is also possible that the Delacour's langur population might have actually increased over the last 3 years, given the fact that we were able to record the presence of juveniles and infants in a number of langur groups (Table 1). The findings emphasize the urgency of implementing immediate and sustained conservation measures to safeguard and potentially facilitate the recovery of this Critically Endangered primate in Kim Bang.

## Author Contributions

**Anh Tuan Nguyen:** conceptualization (lead), formal analysis (lead), investigation (equal), writing – original draft (equal). **Linh Nguyen:** formal analysis (supporting), investigation (equal), writing – original draft (supporting). **Hoang Trinh‐Dinh:** conceptualization (supporting), formal analysis (supporting), investigation (supporting), writing – original draft (equal). **Phong Nguyen:** formal analysis (supporting), investigation (equal), writing – original draft (supporting). **Thanh Nguyen:** conceptualization (supporting), formal analysis (supporting), writing – original draft (supporting). **Minh Le:** conceptualization (supporting), formal analysis (supporting), investigation (supporting), writing – original draft (equal).

## Conflicts of Interest

The authors declare no conflicts of interest.

## Data Availability

All datasets are included in the paper's main text.
